# Insights into the Phylogeny and Evolution of Cold Shock Proteins: From Enteropathogenic *Yersinia* and *Escherichia coli* to Eubacteria

**DOI:** 10.3390/ijms20164059

**Published:** 2019-08-20

**Authors:** Tao Yu, Riikka Keto-Timonen, Xiaojie Jiang, Jussa-Pekka Virtanen, Hannu Korkeala

**Affiliations:** 1 Department of Life Science and Technology, Xinxiang University, Xinxiang 453003, China; 2 Department of Food Hygiene and Environmental Health, University of Helsinki, P.O. Box 66, FI-00014 Helsinki, Finland

**Keywords:** cold shock protein, enteropathogenic *Yersinia*, eubacteria, phylogeny, evolution

## Abstract

Psychrotrophic foodborne pathogens, such as enteropathogenic *Yersinia*, which are able to survive and multiply at low temperatures, require cold shock proteins (Csps). The Csp superfamily consists of a diverse group of homologous proteins, which have been found throughout the eubacteria. They are related to cold shock tolerance and other cellular processes. Csps are mainly named following the convention of those in *Escherichia coli*. However, the nomenclature of certain Csps reflects neither their sequences nor functions, which can be confusing. Here, we performed phylogenetic analyses on Csp sequences in psychrotrophic enteropathogenic *Yersinia* and *E. coli*. We found that representative Csps in enteropathogenic *Yersinia* and *E. coli* can be clustered into six phylogenetic groups. When we extended the analysis to cover Enterobacteriales, the same major groups formed. Moreover, we investigated the evolutionary and structural relationships and the origin time of Csp superfamily members in eubacteria using nucleotide-level comparisons. Csps in eubacteria were classified into five clades and 12 subclades. The most recent common ancestor of Csp genes was estimated to have existed 3585 million years ago, indicating that Csps have been important since the beginning of evolution and have enabled bacterial growth in unfavorable conditions.

## 1. Introduction

Psychrotrophic foodborne pathogens, such as enteropathogenic *Yersinia,* which are able to survive and multiply at low temperatures, pose a risk in modern food production, where cold chains are used to increase the shelf lives of food products [1,2,3]. Cold-induced proteins (Cips) are important for growth at low temperatures and are involved in RNA metabolism, protein folding and the synthesis of membrane lipid A [4]. Cips include, e.g., homologous Csps that are classified together in the Csp family [5]. An abrupt temperature downshift (cold shock) causes a cold shock response in bacteria, during which only a limited number of proteins are induced [6]. Many *csp* genes are highly induced after a temperature downshift, and Csps are known to play a key role in survival after cold shock and in adaptation to low growth temperature [4,6,7,8,9,10,11,12,13,14]. Recent studies have shown that Csps may also aid in other stress responses [5,15,16,17]. Csps are small nucleic acid-binding proteins that have been found in a variety of eubacteria, including psychrophiles, mesophiles and thermophiles [18]. Very few research papers have been published concerning the need for *csp* genes in psychrotrophic *Yersinia* [5,19,20]. Most Csp studies have been conducted using mesophilic *E. coli* [6,10,11,12,13,21,22,23,24,25]. Understanding how the Csps of psychrotrophic bacteria, such as enteropathogenic *Yersinia*, differ from those of mesophilic bacteria is important, for this may represent a common feature of psychrotrophs that distinguishes them from mesophiles. Furthermore, a deeper understanding of the Csps of psychrotrophs could contribute to preventing their growth in refrigerated food or in optimizing the production of their enzyme for biotechnological purposes [26].

The Csps have two conserved RNA-binding motifs, RNP1 and RNP2 [10,27,28], which may be responsible for selective RNA-binding activity [21]. CspA was first identified in *E. coli* [6,11]. So far, a total of nine homologous Csps (CspA-CspI) have been found in *E. coli*, defined by their protein sequences [12]. However, all Csps in *E. coli* are not related to the cold shock response. Only CspA, CspB, CspE, CspG and CspI are generally recognized as cold induced [6,10,11,13,22,29]. CspC and CspE are involved in the regulation of alternative sigma factor σ^S^ and universal stress protein UspA, and in growth [23,24,30]. CspD is induced at the early stationary phase during nutritional starvation [25]. 

Csps are mainly named following the convention of *E. coli* Csps. However, the nomenclature of certain Csps reflects neither their sequences nor functions [5]. For example, of the ten Csps in *Yersinia enterocolitica* subsp. *enterocolitica* 8081, CspA2 (*YE3823*) has the highest amino acid similarity with CspI of *E. coli* [5]. In *Yersinia pseudotuberculosis* IP32953, CspA2 (*YPTB3587*) is also the most similar Csp to CspI of *E. coli* [5]. Moreover, unlike the CspD in *E. coli*, CspD in *Bacillus subtilis* is cold induced [31]. The disconnected nomenclature of Csps in various bacteria can be confusing. With increasing numbers of sequenced bacterial genomes, certain Csps have been found to have no high sequence similarity to any of those in *E. coli* [5,32,33].

In this study, we performed phylogenetic analyses on Csp sequences in enteropathogenic *Yersinia, E. coli* and eventually all Enterobacteriales. Based on the variation in amino acid residues, Csps in Enterobacteriales were divided into six major groups. Moreover, we performed evolutionary analyses on the *csp* genes, and clarified the phylogeny and origin time of Csps in eubacteria. Five clades and twelve subclades were identified in eubacterial Csps. The estimation of the timing of Csp evolution, based on the time to the most recent common ancestor (tMRCA) of *csp* genes in eubacteria, suggests that Csps emerged 3585 million years ago (MYA), with a mean mutation rate of 4.392 × 10^-4^ substitutions per site per million years.

## 2. Results

### 2.1. Phylogeny and Consistency of Csps in Enteropathogenic Yersinia Enterocolitica and Yersinia Pseudotuberculosis

**Figure 1:**
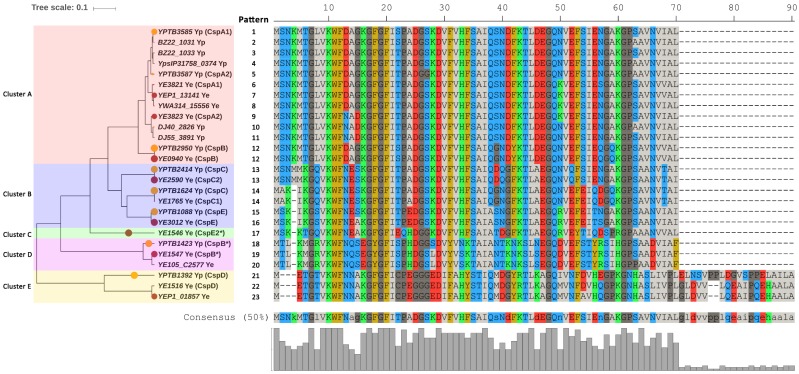
Phylogenetic cluster of Csp sequence patterns in enteropathogenic *Yersinia*. A phylogenetic tree composed of representative Csps of sequence patterns in enteropathogenic *Yersinia*. Circle radii represent the abundance ratios of each Csp pattern in *Y. pseudotuberculosis* (orange) or *Y. enterocolitica* (brown). Most Csps were named using the convention of *E. coli*, if any, except those marked with an asterisk. Histogram shows conservation of Csp sequence.

A total of 447 Csp sequences with explicit gene symbols in *Y. enterocolitica* (*n* = 13) and *Y. pseudotuberculosis* (*n* = 45) strains (Table S1) were retrieved from the Pathosystems Resource Integration Center (PATRIC) database. Twenty-three Csp sequence patterns were identified and separated into five phylogenetic clusters (Figure 1, Table S2). Csps in the same pattern shared 100% amino acid sequence identity with each other. Out of these patterns, 10 patterns were specific for *Y. enterocolitica*, 10 patterns were only found in *Y. pseudotuberculosis* and both species harbored patterns 12, 13 and 14 (Figure 1, Tables S3 and S4). 

The pairwise evolutionary distances between respective Csps of all Csp sequence patterns in *Y. enterocolitica* and *Y. pseudotuberculosis* were calculated based on the phylogenetic tree (Figure 1) and visualized using a heat map (Figure S1). Corresponding homologues of the representative Csps for 12 *Y. enterocolitica* patterns could be identified in the representative Csps of *Y. pseudotuberculosis* patterns. Particularly, Csps in cluster A, in which 12 of 23 patterns were included, were homologous, with a maximum distance of 0.14. The maximum distances of cluster B (four patterns included), cluster D (three patterns included) and cluster E (three patterns included) were 0.27, 0.08 and 0.20 respectively. However, pattern 17 represented by CspE2 (*YE1546*) forms a monophyletic branch (cluster C) separated from the other patterns, and no identical pattern was found in *Y. pseudotuberculosis*.

### 2.2. Phylogeny and Consistency of Csps in E. coli

**Figure 2:**
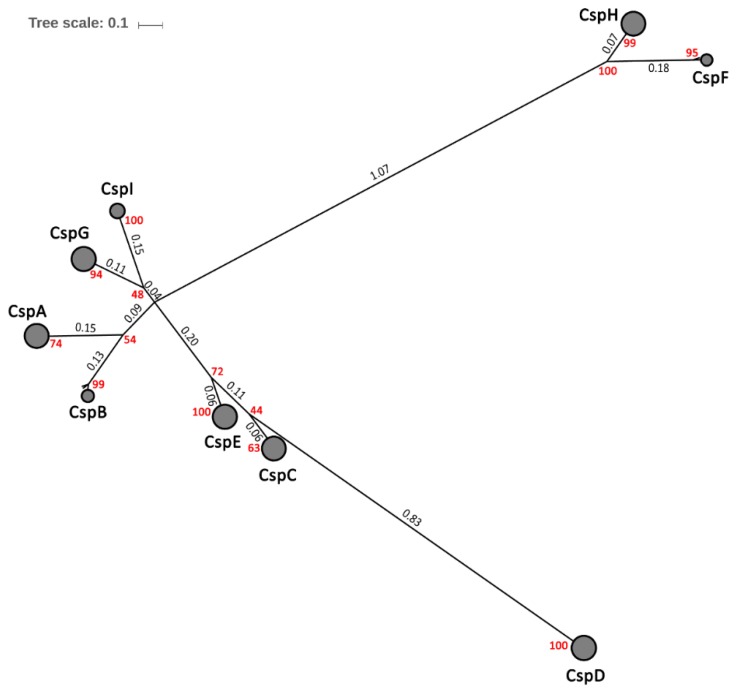
Phylogenetic tree for Csps in *Escherichia coli*. A phylogenetic tree composed on 1013 Csps in 135 *E. coli* strains. Monophyletic nodes have been collapsed and are represented by a circle. The number of Csps in a collapsed node is indicated by the circle radius. Black numbers indicate the evolutionary distances between the main nodes, and red numbers indicate the bootstrap values.

A total of 1013 Csp sequences in 135 *E. coli* strains (Supplementary Data 1) were retrieved from the PATRIC database. The phylogenetic tree showed that Csps were aggregated together into nine discrete regions of the tree, corresponding to nine Csp members in *E. coli*, CspA to CspI (Figure 2). Csps within each paralog were highly conserved across all the *E. coli* strains. In 135 *E. coli* strains, 20 Csp sequence patterns were identified, and the amino acid sequences were highly conserved in each Csp member (Figure S2, Table S5). The sequences of CspA in 135 representative *E. coli* strains were identical, as were the sequences of CspC, CspD and CspE. CspG had two different sequence patterns, whereas CspF, CspH and CspI all had three different patterns. Five sequence patterns were identified in CspB and it therefore appeared to be more polymorphic than other Csp members in *E. coli*. However, only four polymorphic sites at the most were identified between pairs of CspB sequences, and the substitutions were limited in structurally similar amino acids such as phenylalanine and tyrosine, or isoleucine and valine (Figure S2). For convenience, nine Csps in *E. coli* str. K-12 substr. MG1655 were considered representative Csps of *E. coli* species in the following analysis.

The pairwise evolutionary distances between nine representative Csps in *E. coli* were estimated according to the phylogenetic tree presented in Figure 2 and displayed in a distance matrix (Table S6). Of the nine Csp members, CspA, CspB, CspG and CspI were more similar to each other, with a maximum distance of 0.43. CspC and CspE, along with CspF and CspH were homologous Csps with evolutionary distances of 0.23 and 0.25, respectively. However, the CspF/CspH cluster was phylogenetically far from the other Csp members. All distances between CspF/CspH and other Csp members were larger than 1.2, indicating that the CspF/CspH cluster evolves independently. Similar to the CspF/CspH cluster, the minimum distance between CspD and other Csp members was 0.89, suggesting that CspD is also a Csp member evolving independently.

### 2.3. Comparison of Csps in Enteropathogenic Yersinia and E. coli

**Figure 3:**
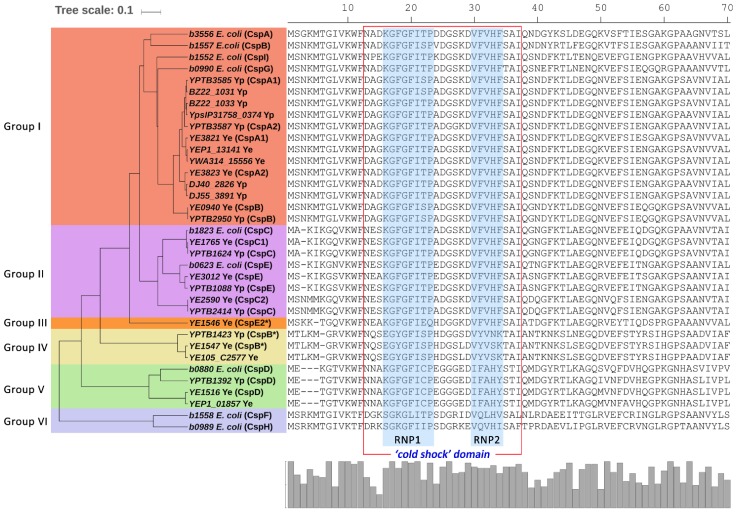
Phylogenetic group of representative Csps in *Escherichia coli* and enteropathogenic *Yersinia*. A phylogenetic tree and alignment of representative Csps in *E. coli* and enteropathogenic *Yersinia.* The phylogenetic groups of representative Csps were represented by color. Conserved RNP motifs of Csps were colored light blue. Most Csps were named using the convention of *E. coli*, if any, except those marked with an asterisk. The alignment is trimmed to the 70th site for better viewing. Histogram shows Csp sequence conservation.

We constructed a phylogenetic tree combining representative Csps in enteropathogenic *Yersinia* and *E. coli* (Figure 3). Based on amino acid sequences, representative Csps in enteropathogenic *Yersinia* and *E. coli* were clustered into six phylogenetic groups (Figure 3 and Figure S3, Table S7). Cold-inducible Csps of *E. coli*, such as CspA, CspB, CspG and CspI, are included in group I, but Csps of *Yersinia* in group I form a monophyletic group separated from *E. coli* Csps. However, the groups still have a close phylogenetic relationship with each other, suggesting that these Csps should belong to one monophyletic group. All CspC and CspE of *Yersinia* and *E. coli* except CspE2 (*YE1546*) formed monophyletic group II, indicating that these Csp members are conserved in enteropathogenic *Yersinia* and *E. coli*. Similarly, in group V, CspDs also formed a monophyletic group. Group III consisted of only one CspE2 pattern identified in *Y. enterocolitica*. CspF and CspH patterns were identified only in *E. coli* and formed a monophyletic group VI far apart from other Csp patterns, with a specific RNP2 sequence (consensus VQVLHIV). Similar to CspF and CspH in *E. coli*, three CspB patterns only identified in *Yersinia* (*YE1547*, *YE105_C2577* and *YPTB1423*) also had a specific RNP2 sequence (consensus VYVSNK), forming monophyletic group IV. 

### 2.4. Phylogenetic Diversity of Csps in Enterobacteriales

**Figure 4:**
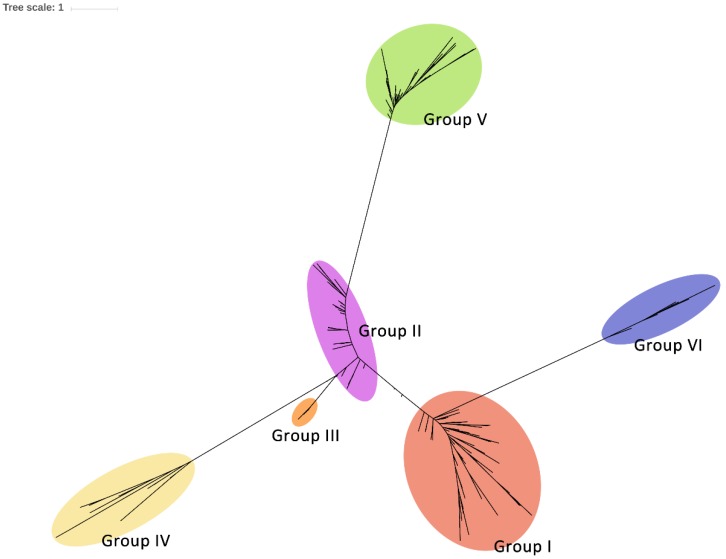
Phylogenetic tree for Csps in Enterobacteriales. A phylogenetic tree composed on 322 non-redundant Csps in 104 Enterobacteriales strains. The phylogenetic groups of Csps are represented by color. The complete tree with full bootstrap values is available in Newick format as Supplementary Data 2.

We assessed an alignment of 322 non-redundant Csp sequences in 104 Enterobacteriales strains (Table S8) to gain a more comprehensive view on Csp phylogeny. Tree reconstructions reinforced our previous results of clustering Csps into six groups, at least in Enterobacteriales (Figure 4). We also investigated the phylogeny of the 322 Csps using just their cold shock domain (CSD) sequences. The underlying topology of the latter tree (Figure S4) very closely matched the topology of the former tree (Figure 4), and showed similar phylogenetic grouping, suggesting that the phylogeny of Csps could be determined by their CSD sequences in Enterobacteriales.

### 2.5. Clarification of Phylogeny and Origin Time of Csp Genes in Eubacteria 

**Figure 5:**
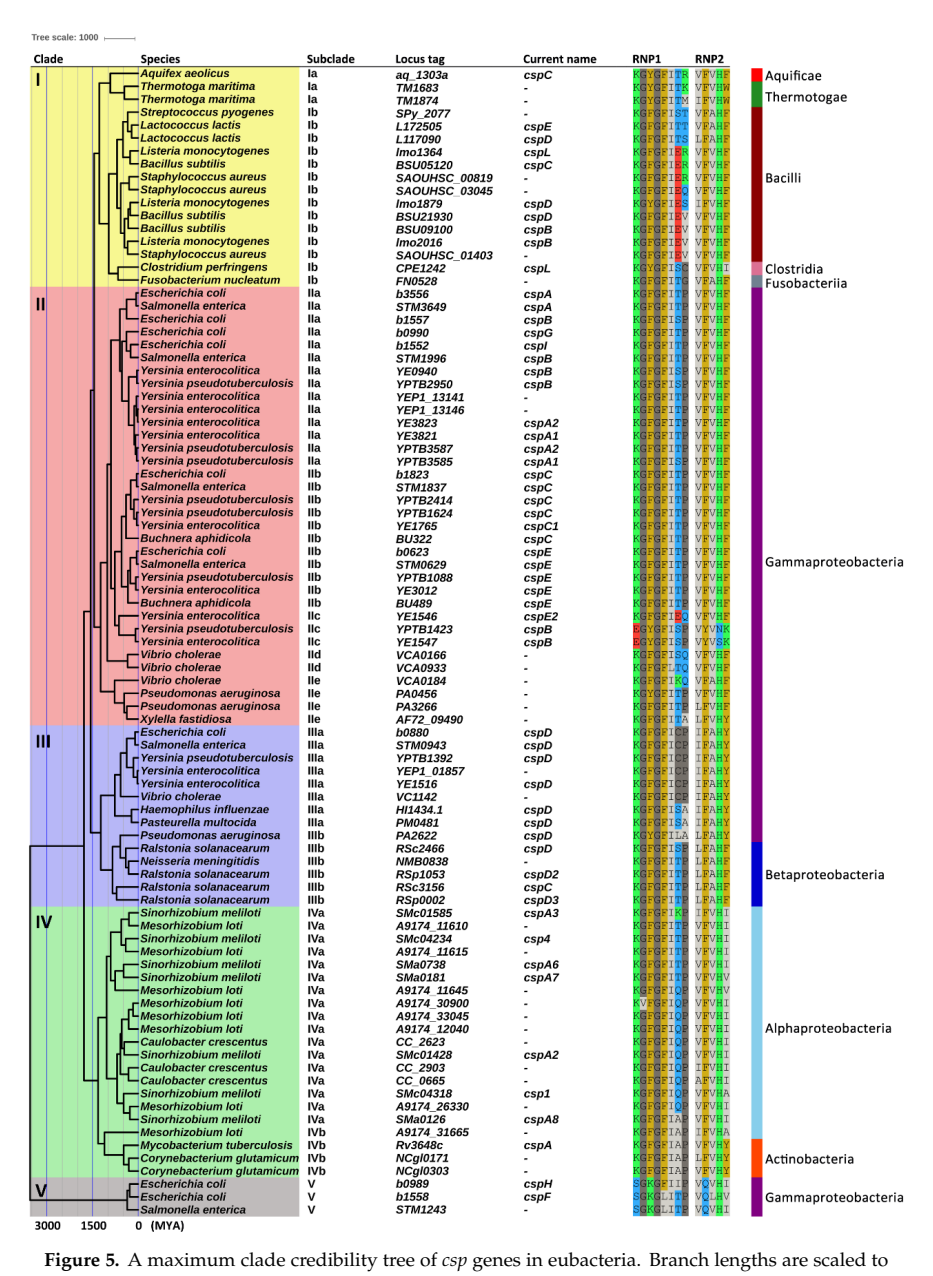
A maximum clade credibility tree of *csp* genes in eubacteria. Branch lengths are scaled to years. Branch lengths are given as millions of years ago (MYA). The phylogenetic clades of Csps are represented by color. The bacterial class of the organism containing each *csp* gene sequence is represented by the color of the strip.

To evaluate the phylogeny and origin time of Csp in eubacteria, we constructed maximum clade credibility (MCC) tree in BEAST for 89 *csp* gene sequences from 26 bacterial taxa (Figure 5). Phylogenetic analysis of these 89 Csps showed that Csps in eubacteria could be classified into five clades and 12 subclades (Table 1). 

In addition, we estimated the tMRCA of all known *csp* genes in eubacteria to be 3585 MYA (95% highest posterior density (HPD) interval: 2132 to 5554 MYA), with a mean mutation rate of 4.392 × 10^−4^ substitutions per site per million years (95% HPD interval: 4.056 × 10^−4^ to 4.747 × 10^−4^). To validate the reliability of this estimation, we also collected 26 gene sequences of 16S RNA from the 26 bacterial taxa, and calculated the tMRCA (3224 MYA with a 95% HPD interval from 2127 to 4696 MYA) and mean mutation rate (1.333 × 10^−4^ substitutions per site per million years with a 95% HPD interval from 9.550 × 10^−5^ to 1.710 × 10^−4^ substitutions per site per million years) of 16S rRNA genes in eubacteria using the same method. Our results are consistent with previous studies [34,35].

## 3. Discussion

In the present study, we investigated the diversity and consistency of Csp sequences in enteropathogenic *Yersinia, E. coli* and Enterobacteriales. A total of 23 Csp sequence patterns were identified by analyzing 447 Csps from 58 enteropathogenic *Yersinia* strains. Pattern 17 represented by CspE2 (*YE1546*) formed a monophyletic branch separated from other patterns, and no identical pattern was found in *Y. pseudotuberculosis*, suggesting this Csp pattern may perform certain specific functions in *Y. enterocolitica.* According to Figure 1, CspD patterns (*YE1516* and *YEP1_01857* in *Y. enterocolitica* and *YPTB1392* in *Y. pseudotuberculosis*) formed a monophyletic clade separate from other Csp members in enteropathogenic *Yersinia*, indicating that CspD is an independent Csp member. Noteworthily, three CspB patterns (*YE1547*, *YE105_C2577* in *Y. enterocolitica* and *YPTB1423* in *Y. pseudotuberculosis*) also formed a monophyletic clade, which separated from other CspB and CspA patterns in enteropathogenic *Yersinia*. Likewise, CspE2 (*YE1546* in *Y. enterocolitica*) also separated from other CspE patterns (patterns 15 and 16). These results confirm the perception that the currently used nomenclature is not appropriate for all Csps.

Moreover, 23 Csp sequence patterns in enteropathogenic *Yersinia* and nine representative Csps in *E. coli* were classified into six phylogenetic groups due to the alignment of their amino acid sequences (Figure 3). In group I, Csps from enteropathogenic *Yersinia* (patterns one to 12 in Figure 1) were phylogenetically closer to cold-inducible Csps (CspA, CspB, CspG and CspI) [18] than other Csps in *E. coli*. One of the unique features of *cspA*, *cspB*, *cspG* and *cspI* in *E. coli* is the unusually long (159, 160, 155 and 145 bases, respectively) 5′ untranslated region (5′-UTR), which contains a highly conserved unique sequence called the cold box [14,18]. Interestingly, the highly similar sequences were also identified in the 5′-UTR of enteropathogenic *Yersinia* Csps in group I. Another unique feature of cold-inducible *csp* genes of *E. coli* in group I is a highly conservative sequence located 14 bases downstream of the initiation codon, which has been termed the downstream box [10,18]. This element was also present in the *csp* genes of enteropathogenic *Yersinia* that were phylogenetically close to the cold-inducible group I Csps of *E. coli* (Figure 3 and Figure S5). These findings indicate that Csps from psychrotrophic enteropathogenic *Yersinia* in group I may also connect with cold-adaptation functions. However, the ability of enteropathogenic *Yersinia* to grow at near-zero temperatures does not appear to depend on these *csp* genes. In mesophilic *E. coli,* although CspE can complement the cold-sensitive phenotype of the quadruple *△cspA△cspB△cspG△cspE* deletion strain [29], CspE was classified as group II together with CspC (Figure 3), considering high similarity in the protein sequence and roles in transcription regulation [23,36]. Furthermore, no cold box or downstream box was identified in *cspE* in *E. coli*, demonstrating it should not be classified as group I.

Our study defined two *Yersinia*-specific Csp groups. Group III is represented by *YE1546* in *Y. enterocolitica*, and Group IV is represented by *YE1547* and *YE105_C2577* in *Y. enterocolitica* and *YPTB1423* in *Y. pseudotuberculosis*. *YPTB1423* is upregulated during growth at low temperatures in *Y. pseudotuberculosis* IP32953 [38]. However, their role in the cold adaptation of enteropathogenic *Yersinia* should be clarified.

Group V is mainly formed by CspDs in *E. coli* and enteropathogenic *Yersinia*. Stationary-phase-induced CspD (b0880) in *E. coli* [25] may evolve independently due to the large phylogenetic distance of CspD from other Csps (Figure 2). Group VI is represented by CspF (b1558) and CspH (b0989) in *E. coli*. Functions of Csps in this group are still unknown. CspF and CspH in *E. coli* are probably also independent in evolution, considering the phylogenetic distance of CspF/CspH from other Csps. The orthologs of CspF or CspH in *E. coli* could not be found in Csps in *Yersinia*.

The classification of Csps into six groups was still valid when extending Csps in the order of Enterobacteriales (Figure 4), but not in eubacteria. Phylogenetic analysis of the 89 Csps from 26 taxa suggested that Csps in eubacteria could be classified into five clades and 12 subclades (Figure 5, Table 1). Homologues of CspF and CspH in *E. coli* (clade V, in which group VI of Enterobacteriales Csps is included) were most distantly related to all the other known Csp homologues. Moreover, they did not express one of the characteristic features of other Csp homologues, i.e., a high aromatic amino acid content, which is an essential feature required for the RNA-binding role of Csps [12]. CspF and CspH in *E. coli* only have three and four aromatic residues, respectively, in contrast to approximately eight aromatic residues found in other Csp homologues [12]. In addition to the distinctive assumed RNP sequences, a substantial evolutionary divergence has occurred between CspF/CspH homologues (clade V) and other Csp homologues (clades I to IV). In clade I, subclade Ia is hyperthermophilic and bacteria-specific. Csps classified as subclade Ib mainly existed in gram-positive bacteria, and some are involved in the regulation of cold and osmotic stress tolerance, virulence, cellular aggregation and flagella-based motility [7,15]. Clade II is the largest group that includes most of the ‘classical Csps’, consisting of subclades IIa to IIe. A subset of the Csps in this clade are involved in cold tolerance and transcriptional regulation [6,11,12,18,23,29,36]. Most known cold-inducible Csps are classified as subclade IIa, in which group I of Enterobacteriales Csps is included. Many transcription regulation-related Csps belong to subclade IIb, in which Enterobacteriales Csp group II is included. Subclade IIc is *Yersiniaceae*-specific, including groups III and IV of Enterobacteriales Csps (Figure 4 and Figure 5). Csp functions are unknown in subclades IId and IIe. Clade III contains *cspD* genes, which were separated from the other *csp* genes in gram-negative bacteria. The representative Csp of clade III is CspD (*b0880*) in *E. coli*, which participates in responses to nutrient stress [25,37]. Group V of Enterobacteriales Csps is included in subclade IIIa (Figure 4 and Figure 5), and it is noteworthy that the currently named *cspD* genes in gram-positive bacteria were not included in this clade. Furthermore, this CspD clade of gram-negative bacteria only included Betaproteobacteria and Gammaproteobacteria, indicating that CspD is specific for these two classes. Generally, *csp* genes in the same bacterial class were clustered together, suggesting that *csp* genes represented in each class evolved from a single ancestral Csp homologue, except *cspD* genes in clade III. Clade IV contains Csps from Alphaproteobacteria and Actinobacteria. The functions of Csps in this clade are unclear.

Divergence time estimates for the major groups of eubacteria range between 2.5 and 3.2 billion years ago [35]. However, CSD-containing proteins have been found in all three domains of life [39,40,41,42,43], suggesting that a CSD-like protein was present before the divergence of bacteria and eukarya/archaea. Thus, an ancestral Csp may be found at the beginning of single-cell evolution, approximately 3.5 billion years ago [37]. This inference is supported by our evaluation (Figure 5). Csps are small and conserved proteins with large numbers of homologues. However, the nomenclature of *csp* genes is misleading and limited, due to being first found in cold shock studies. In fact, *csp* genes are probably general stress response or adaptation genes. Csps play a more general role than their sole implication in cold adaptation. They have been important tools in bacterial adaption to various, often disadvantageous, conditions. The presence of Csps in Archaea [39,40] and the earliest-diverging hyperthermophilic bacteria [42,43] also indicates that bacteria may originally have needed them to survive extreme conditions, not just cold. Compared with the synonymous mutation rate (4.5 × 10^-3^ substitutions per site per million years) in *E. coli* [34], the low mean mutation rate (4.392 × 10^-4^ substitutions per site per million years) of *csp* genes in eubacteria has limited their heterogeneity. Csps have not been adapting to change rapidly and the evolution of *csp* genes seems to be slow compared to many other genes.

## 4. Materials and Methods

### 4.1. Selection of Csp Sequences from Databases 

All the Csp sequence data for the eubacteria used in our work were collected from the Pathosystems Resource Integration Center (PATRIC; https://www.patricbrc.org/) [44] and the National Center for Biotechnology Information (NCBI; https://www.ncbi.nlm.nih.gov/). In *E. coli* and enteropathogenic *Yersinia*, Csp sequences are 65 to 75 [5,31] and 69 to 87 (this study) amino acids (aa) in length, respectively. Thus, the amino acid sequences annotated as cold shock proteins but shorter than 65 aa or longer than 87 aa were excluded from further analyses.

### 4.2. Protein Clustering, Alignment and Phylogeny 

To generate non-redundant datasets for the phylogenetic analyses, 447 Csp sequences of enteropathogenic *Yersinia* (104 Csps from 13 *Y. enterocolitica* strains and 343 Csps from 45 *Y. pseudotuberculosis* strains), 1013 Csp sequences from 135 *E. coli* and 534 Csp sequences from 104 Enterobacteriales were clustered as three separate datasets at the 100% identity level using CD-Hit v4.5.4 [45]. Nine Csps in *E. coli* str. K-12 substr. MG1655 were considered as representative Csps of *E. coli* species in the following analyses. For Csp phylogeny, the MAFFT v7 (https://mafft.cbrc.jp/alignment/server/index.html) [46] with G-INS-i strategy (Unalignlevel 0.3, “leave gappy regions” set, and other default parameters) was used for all alignments. Maximum likelihood phylogenetic trees of aligned proteins were inferred with RAxML v8.2.9 [47], with a protein-specific amino-acid substitution model identified using RAxML (PROTGAMMAAUTO); tree topologies were checked by 100 bootstrapping replicates. iTOL v3 (http://itol.embl.de/) [48] was used to visualize the trees.

### 4.3. Estimation of Substitution Rate and Origin Time of Csp and 16S rRNA 

The gene sequences of 89 Csps and 26 16S rRNAs from 26 taxa in eubacteria were obtained from PATRIC, respectively. The GTR+I+G4 and GTR+F+I+G4 substitution models were selected as the best-fit nucleotide substitution models for Csp and 16S rRNA respectively, by ModelFinder, which is included in IQ-TREE v1.5.4 [49]. A Bayesian framework using BEAST v2.4.7 and associated software (Beauti v2.4.7, Tracer v1.6.0, LogCombiner v2.4.7, TreeAnnotator v2.4.7) [50] were used to date the phylogeny and estimate the mutation rate for Csp and 16S rRNA in eubacteria, respectively. Analyses used the GTR model of nucleotide substitution with four discrete gamma-distributed rate categories and were run using a log-normal relaxed clock and the coalescent constant population model of speciation. The default settings were used to estimate priors for all other parameters. A run was also performed without data to assess prior distributions and allow comparisons with posterior distributions. The Markov chain Monte Carlo (MCMC) in BEAST was run for 100 million generations and sampled every 10,000 generations. Four replicate runs were performed to check for convergence of the MCMC and combined together after discarding 10 million generations from each run as burn-in. We extracted the clock rate and tMRCA estimate, and their distributions, with Tracer v1.6.0. Finally, the MCC tree was built using TreeAnnotator v1.6.0 with 10% burn-in and visualized in Figtree v1.4.3 (http://tree.bio.ed.ac.uk/software/figtree/) and iTOL v3.

### 4.4. Molecular Clock Calibration 

The present phylogenetic analyses showed that CspD proteins belonging to the CspD family formed a monophyletic group separated from other Csp members in Enterobacteriales (Figure 4), assuming that CspD evolves independently of other Csps. We calibrated the node that corresponded with the presumed divergence event between *cspD* genes in *E. coli* and *Salmonella enterica* (*b0880* and *STM0943*). To ensure the reliability of this estimation, we calibrated another node, which corresponded to the divergence event of *cspD* genes in *Haemophilus influenzae* and *Pasteurella multocida* (*HI1434.1* and *PM0481*). The priors for these nodes were assigned normal distribution with standard deviation (s.d.) based on the divergence time of *E. coli* and *S. enterica, H. influenzae* and *P. multocida,* respectively. The divergence event of *b0880*–*STM0943* was calibrated by setting the prior for that node to 102 MYA with a s.d. of 3.0, while *HI1434.1*–*PM0481* divergence was calibrated at 220 MYA with a s.d. of 11.0 [35].

The reported credible intervals around estimates are 95% HPD intervals. Ages of various nodes were estimated from the resulting tree, using the 95% HPD as an estimate of error for the age of each node. When reporting the mutation rate from a relaxed-clock model, the mean rate (mean of the rates of each branch weighted by the time length of the branch) is given.

### 4.5. Data Availability

The log files generated by BEAST and phylogenetic trees are available online (https://github.com/fffish8888/Csp-2018). The authors declare that the other main data supporting the findings of this study are available within this Article and in the Supplementary Information files. 

Other data that support the findings of this study are available from the corresponding author upon request.

## Figures and Tables

**Table 1:** Cold shock protein gene clades in eubacteria.

Clade ^1^	Subclade ^1^	Representative *csp* Gene(s)	Currently Used Name(s) ^2^	RNP1 ^3^	RNP2 ^3^	Known Function of Csps and Occurrence in Bacteria
I	Ia	*aq_1303a*	*cspC*	KGYGFITX	VIFVHWF	Exists in the hyperthermophilic bacterium.
	Ib	*Lmo2016, BSU05120, lmo1879, L172505, CPE1242*	*cspB, cspC, cspD, cspE, cspL*	KGFYGFIXX	XFVAHFI	Involves in regulation of cold and osmotic stress tolerance, virulence, cellular aggregation and flagella-based motility [7,15]; mainly exists in gram-positive bacteria.
II	IIa	*b3556, b1557, b0990, b1552*	*cspA, cspB, cspG, cspI*	KGFGFITSP	VFVHF	Cold-inducible [6,11,18]; exists in Gammaproteobacteria.
	IIb	*b1823, b0623*	*cspC, cspE*	KGFGFITP	VFVHF	Involves in cold adaptation (CspE) [29], transcriptional regulation and/or chromosome condensation [12,23,36]; exists in Gammaproteobacteria.
	IIc	*YPTB1423, YE1546*	*cspB, cspE2*	KEGFYGFIESQP	VFYVXFK	Involves in stress response *in vivo* [17]; mainly exists in *Yersiniaceae*.
	IId	*VCA0166*	*-*	KGFGFILSTQ	VFVHF	Exists in *Vibrionaceae*.
	IIe	*PA0456*	*-*	KGFYGFIKTX	VLFVAHFY	Exists in Gammaproteobacteria (not including Enterobacteriales).
III	IIIa	*b0880*	*cspD*	KGFGFICSPA	IFAHY	Plays a role in the nutrient-stress response [25,37]; exists in Gammaproteobacteria.
	IIIb	*RSp1053, RSc3156*	*cspD2, cspC*	KGFYGFIXPA	LFAHFY	Mainly exists in Betaproteobacteria.
IV	IVa	*SMc04318, SMc04234*	*csp1, csp4*	KGVFGFIXP	XFVHX	Exists in Alphaproteobacteria.
	IVb	*Rv3648c*	*cspA*	KGFGFIAP	XFVHYA	Mainly exists in Actinobacteria.
V	V	*b0989, b1558*	*cspF, cspH*	SGKGFLIITP	VQVLHIV	Function unknown [10]; only exists in *Enterobacteriaceae*.

^1^ Clustering of Csps in eubacteria is based on the maximum clade credibility tree presented in Figure 5. ^2^ Hyphen indicates a *csp* gene with no current name. ^3^ RNA-binding motif.

## Supplementary Materials

Supplementary materials can be found at https://www.mdpi.com/1422-0067/20/16/4059/s1.

## Abbreviations

5′-UTR	5′ untranslated region
aa	amino acids
BEAST	Bayesian Evolutionary Analysis Sampling Trees
Cips	Cold-induced proteins
CSD	Cold shock domain
CSPs	Cold shock proteins
HPD	Highest posterior density
MAFFT	Multiple Alignment using Fast Fourier Transform
MCMC	Markov chain Monte Carlo
MCC	Maximum clade credibility
MYA	Million years ago
NCBI	National Center for Biotechnology Information
PATRIC	Pathosystems Resource Integration Center
RAxML	Randomized Axelerated Maximum Likelihood
s.d.	standard deviation
tMRCA	time to the most recent common ancestor
