# Supplementary material for: Insights into the Phylogeny and Evolution of Cold Shock Proteins: From Enteropathogenic Yersinia and Escherichia coli to Eubacteria
Source: Int J Mol Sci. 2019 Aug 20;20(16):4059. doi: 10.3390/ijms20164059 (PMC6719143; doi:10.3390/ijms20164059)
Supplement: Supplementary file 1 [file ijms-20-04059-s001.zip › supplementary information-ijms-566477.pdf]

Insights into the phylogeny and evolution of cold shock proteins: from enteropathogenic *Yersinia* and *Escherichia coli* to eubacteria

Tao Yu [1,2,*], Riikka Keto-Timonen [2], Xiaojie Jiang [2], Jussa-Pekka Virtanen [2], Hannu Korkeala [2]

[1] Department of Life Science and Technology, Xinxiang University, 453003 Xinxiang, China;
[2] Department of Food Hygiene and Environmental Health, University of Helsinki, FI-00014 Helsinki University, Finland; riikka.keto-timonen@helsinki.fi (R.K.-T.); xiaojie.jiang@helsinki.fi (X.J.); jussa-pekka.virtanen@helsinki.fi (J.-P.V.); hannu.korkeala@helsinki.fi (H. K.)
* Correspondence: yutao@xxu.edu.cn (T.Y.)

Table of Contents

1. Supplementary Figures
2. Supplementary Tables

1. Supplementary Figures

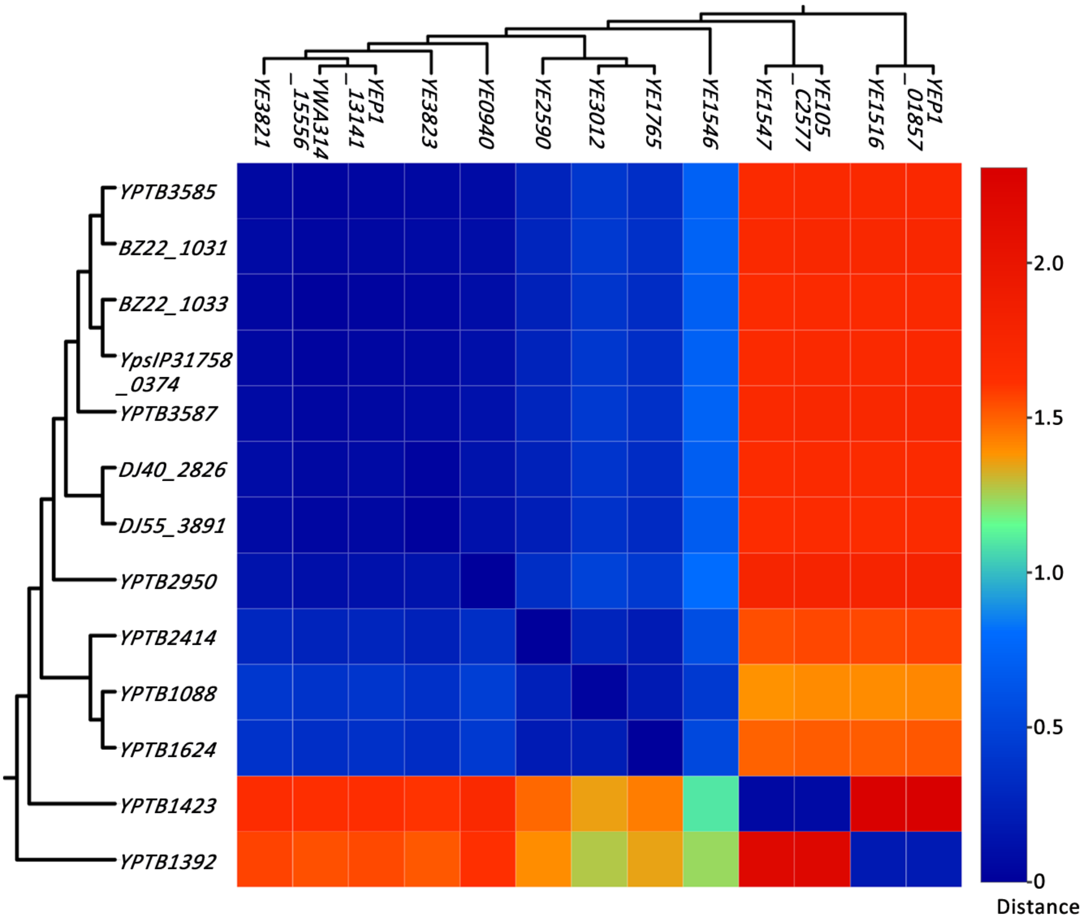

Figure S1. Heat map and hierarchical cluster showing the pairwise similarities between representative Csps in enteropathogenic *Yersinia*. Cool colours (blue, green) indicate similar Csp pairs. Warm colours (red, yellow) indicate dissimilar Csp pairs.

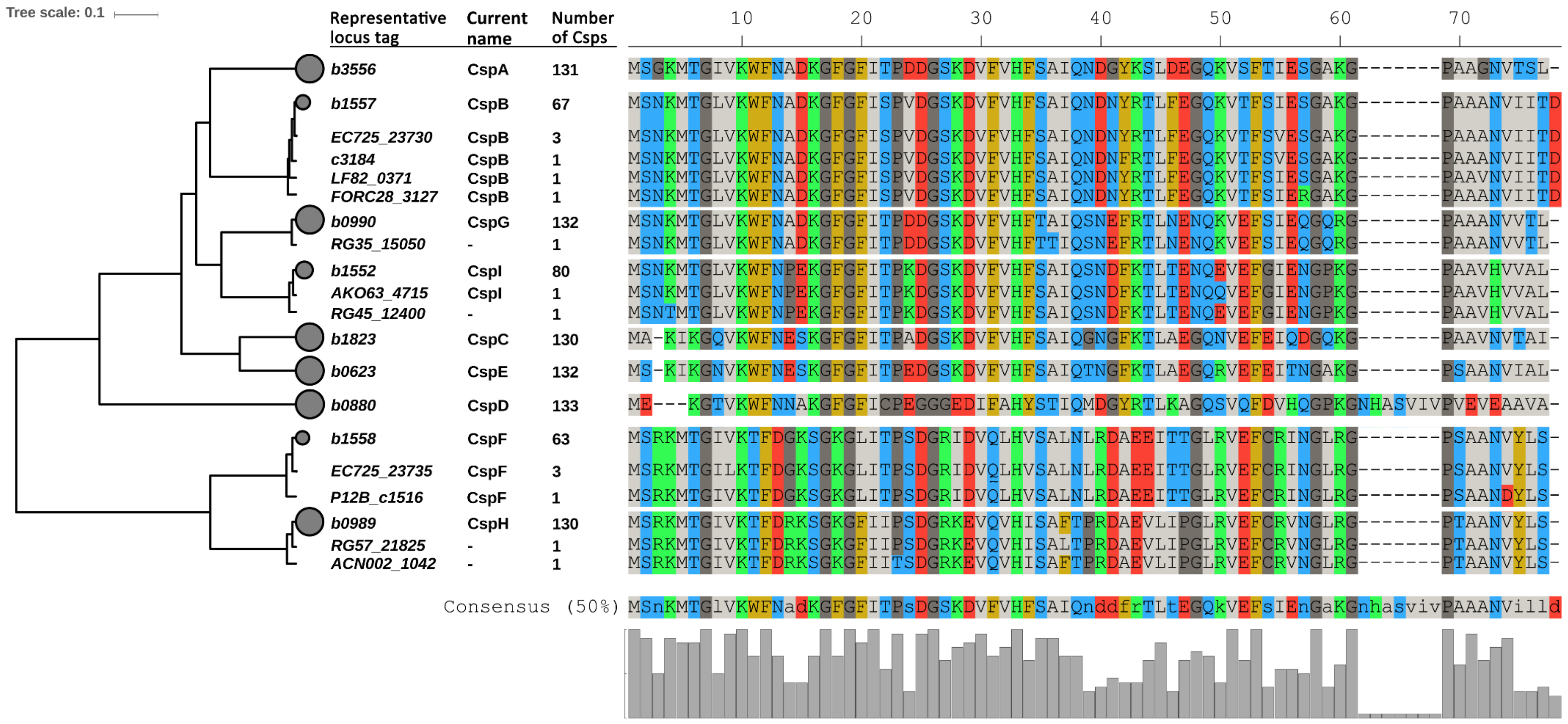

Figure S2. Phylogenetic tree for 20 Csp sequence patterns in *Escherichia coli*. A phylogenetic tree composed on 20 Csp sequence patterns in 135 *E. coli* strains. Monophyletic nodes have been collapsed and are represented by circles. The number of Csp in a collapsed node is indicated by the circle radius. Hyphens indicate *csp* genes with no current name. The histogram shows Csp sequence conservation.

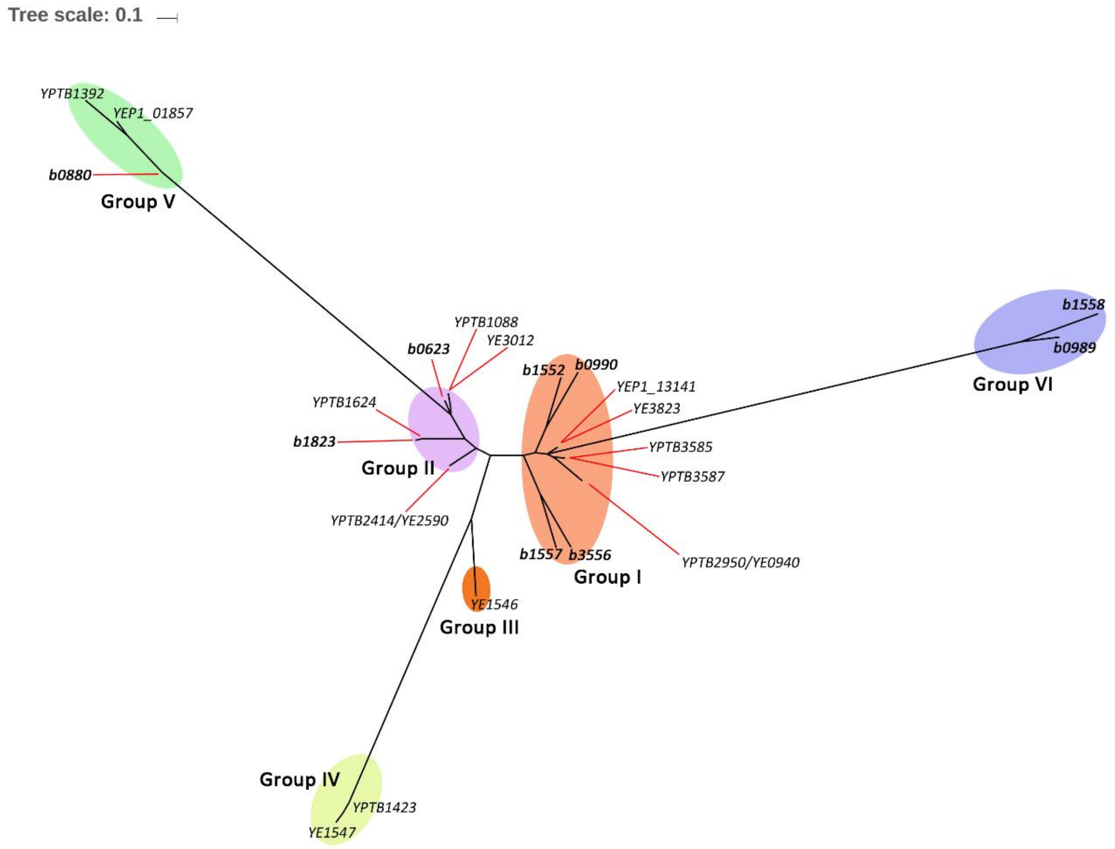

Figure S3. Phylogenetic tree for representative Csps in enteropathogenic *Yersinia* and *Escherichia coli*. A phylogenetic tree composed of the representative Csps in foodborne pathogenic *Yersinia* and *E. coli* (Abundance ratio > 0.3). The phylogenetic groups of representative Csps are represented by colour. The complete tree with full bootstrap values is available in Newick format as Supplementary Data 3.

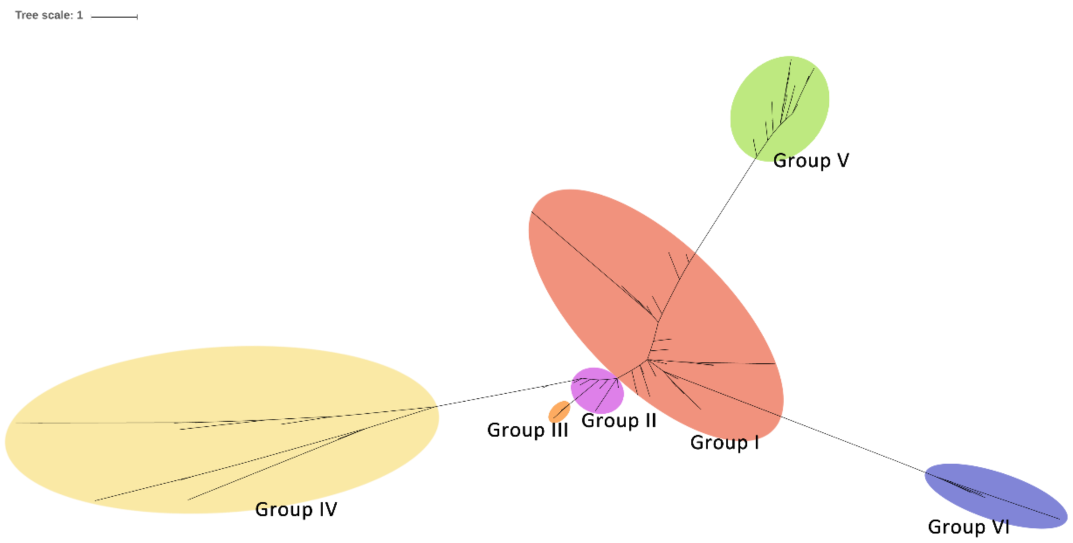

Figure S4. Phylogenetic tree for all Csps in Enterobacteriales. A phylogenetic tree composed on 322 non-redundant Csps in Enterobacteriales, using cold shock domain sequences. The phylogenetic groups of Csps are represented by colour. The complete tree with full bootstrap values is available in Newick format as Supplementary Data 4.

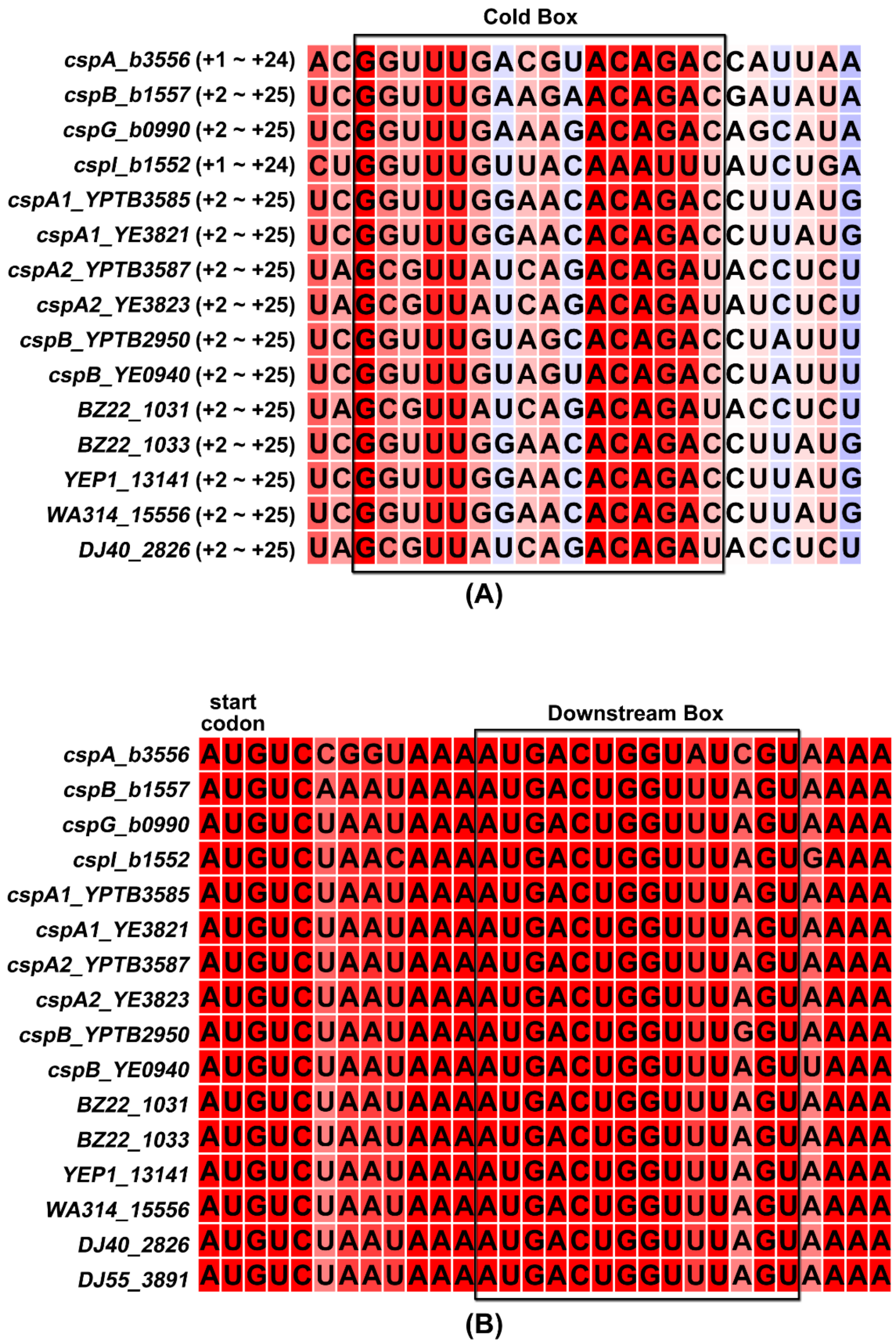

Figure S5. Messenger RNA Sequence comparison of four cold-inducible Csps in *E. coli* and putative cold-inducible *csp* genes in enteropathogenic *Yersinia*. Messenger RNA Sequence alignment of the 5′-UTR (A) and the first 10 codon nucleotides (B) of cold-inducible *cspA* (*b3556*), *cspB* (*b1557*), *cspG* (*b0990*) and *cspI* (*b1552*) in *E. coli* and putative cold-inducible *csp* genes in enteropathogenic *Yersinia* (DJ55_3891 was not included in (A) due to the absence of 5′-UTR sequence information). The highly homologous sequences (cold box and downstream box) are boxed and labeled above the boxes. Red indicates conservative sites and blue indicates unconservative sites.

2. Supplementary Tables

Table S1. Information of Csps in *Yersinia enterocolitica* and *Yersinia pseudotuberculosis*

Species	No. of named Csp	No. of Strain	Representative strain
Y. enterocolitica subsp. *palearctica*	87	11	*Y. enterocolitica* subsp. *palearctica* YE-P1
Y. enterocolitica subsp. *enterocolitica*	17	2	*Y. enterocolitica* subsp. *enterocolitica* 8081
Y. pseudotuberculosis	343	45	*Y. pseudotuberculosis* IP 32953
Total	447	58	

Table S2. Information for each Csp pattern in enteropathogenic *Yersinia.*

Pattern [1]	Cluster	Species	Representative locus tag	Currently used name [2,3]	Abundance ratio [4]
1	A	*Y. pseudotuberculosis*	*YPTB3585*	*cspA1*	0.84
2	A	*Y. pseudotuberculosis*	*BZ22_1031*	*cspG*	0.07
3	A	*Y. pseudotuberculosis*	*BZ22_1033*	*cspG*	0.07
4	A	*Y. pseudotuberculosis*	*YpsIP31758_0374*	-	0.09
5	A	*Y. pseudotuberculosis*	*YPTB3587*	*cspA2*	0.31
6	A	*Y. enterocolitica*	*YE3821*	*cspA1*	0.15
7	A	*Y. enterocolitica*	*YEP1_13141*	-	0.77
8	A	*Y. enterocolitica*	*YWA314_15556*	-	0.15
9	A	*Y. enterocolitica*	*YE3823*	*cspA2*	0.77
10	A	*Y. pseudotuberculosis*	*DJ40_2826*	*cspG*	0.16
11	A	*Y. pseudotuberculosis*	*DJ55_3891*	*cspG*	0.13
12	A	*Y. pseudotuberculosis*	*YPTB2950*	*cspB*	1.00
12	A	*Y. enterocolitica*	*YE0940*	*cspB*	1.00
13	B	*Y. pseudotuberculosis*	*YPTB2414*	*cspC*	0.96
13	B	*Y. enterocolitica*	*YE2590*	*cspC2*	1.00
14	B	*Y. pseudotuberculosis*	*YPTB1624*	*cspC*	1.02
14	B	*Y. enterocolitica*	*YE1765*	*cspC1*	0.15
15	B	*Y. pseudotuberculosis*	*YPTB1088*	*cspE*	1.00
16	B	*Y. enterocolitica*	*YE3012*	*cspE*	1.00
17	C	*Y. enterocolitica*	*YE1546*	*cspE2*	1.00
18	D	*Y. pseudotuberculosis*	*YPTB1423*	*cspB*	0.98
19	D	*Y. enterocolitica*	*YE1547*	*cspB*	0.92
20	D	*Y. enterocolitica*	*YE105_C2577*	-	0.08
21	E	*Y. pseudotuberculosis*	*YPTB1392*	*cspD*	1.00
22	E	*Y. enterocolitica*	*YE1516*	*cspD*	0.15

Table S2. Continued.

**Pattern** [1]	**Cluster**	**Species**	**Representative locus tag**	**Currently used name** [2,3]	**Abundance ratio** [4]
23	E	*Y. enterocolitica*	*YEP1_01857*	-	0.85

[1] Csps with same amino acid sequence.

[2] Naming convention as in *E. coli,* with the exception of Csps in cluster C and D.

[3] Hyphen indicates a *csp* gene with no current name.

[4] Abundance ratio represents Csp number per strain for each Csp pattern.

Table S3. Consistency analysis of 104 Csp sequences in *Yersinia enterocolitica*

Pattern	Pattern size	Representative locus tag	Currently used name [1,2]	Length (aa)
6	2	*YE3821*	*cspA1*	70
7	10	*YEP1_13141*	-	70
8	2	*YWA314_15556*	-	70
9	10	*YE3823*	*cspA2*	70
12	13	*YE0940*	*cspB*	70
13	13	*YE2590*	*cspC2*	70
14	2	*YE1765*	*cspC1*	69
16	13	*YE3012*	*cspE*	69
17	13	*YE1546*	*cspE2* [3]	69
19	12	*YE1547*	*cspB* [3]	69
20	1	*YE105_C2577*	-	69
22	2	*YE1516*	*cspD*	85
23	11	*YEP1_01857*	-	85

[1] Naming convention as in *E. coli* except *YE1546* and *YE1547*.

[2] Hyphen indicates a *csp* gene with no current name.

[3] Nomenclature is inappropriate.

Table S4. Consistency analysis of 343 Csp sequences in *Yersinia pseudotuberculosis*

Pattern	Pattern size	Representative locus tag	Currently used name [1,2]	Length (aa)
1	38	*YPTB3585*	*cspA1*	70
2	3	*BZ22_1031*	-	70
3	3	*BZ22_1033*	-	70
4	4	*YpsIP31758_0374*	-	70
5	14	*YPTB3587*	*cspA2*	70
10	7	*DJ40_2826*	-	70
11	6	*DJ55_3891*	-	70
12	45	*YPTB2950*	*cspB*	70
13	43	*YPTB2414*	*cspC*	70
14	46	*YPTB1624*	*cspC*	69
15	45	*YPTB1088*	*cspE*	69
18	44	*YPTB1423*	*cspB* [3]	69
21	45	*YPTB1392*	*cspD*	87

[1] Naming convention as in *E. coli* except *YPTB1423.*

[2] Hyphen indicates a *csp* gene with no current name.

[3] Nomenclature is inappropriate.

Table S5. Consistency analysis of Csp sequences in 135 *Escherichia coli* strains

Pattern	Pattern size	Representative *csp* gene	Currently used name[1]	Length (aa)
1	131	*b3556*	*cspA*	70
2	67	*b1557*	*cspB*	71
3	3	*EC725_23730*	*cspB*	71
4	1	*c3184*	*cspB*	71
5	1	*LF82_0371*	*cspB*	71
6	1	*FORC28_3127*	*cspB*	71
7	130	*b1823*	*cspC*	69
8	133	*b0880*	*cspD*	74
9	132	*b0623*	*cspE*	69
10	63	*b1558*	*cspF*	70
11	3	*EC725_23735*	*cspF*	70
12	1	*P12B_c1516*	*cspF*	70
13	132	*b0990*	*cspG*	70
14	1	*RG35_15050*	-	70
15	130	*b0989*	*cspH*	70
16	1	*RG57_21825*	-	70
17	1	*ACN002_1042*	-	70
18	80	*b1552*	*cspI*	70
19	1	*AKO63_4715*	*cspI*	70
20	1	*RG45_12400*	-	70

[1] Hyphen indicates a *csp* gene with no current name.

Table S6. Evolutionary distance between nine representative Csps in *Escherichia coli*

	**CspA**	**CspB**	**CspC**	**CspD**	**CspE**	**CspF**	**CspG**	**CspH**	**CspI**
**CspA**	0	0.28	0.61	1.38	0.50	1.49	0.39	1.38	0.43
**CspB**	0.28	0	0.59	1.36	0.48	1.47	0.37	1.36	0.41
**CspC**	0.61	0.59	0	0.89	0.23	1.62	0.52	1.51	0.56
**CspD**	1.38	1.36	0.89	0	1.00	2.39	1.29	2.28	1.33
**CspE**	0.50	0.48	0.23	1.00	0	1.51	0.41	1.40	0.45
**CspF**	1.49	1.47	1.62	2.39	1.51	0	1.40	0.25	1.44
**CspG**	0.39	0.37	0.52	1.29	0.41	1.40	0	1.29	0.26
**CspH**	1.38	1.36	1.51	2.28	1.40	0.25	1.29	0	1.33
**CspI**	0.43	0.41	0.56	1.33	0.45	1.44	0.26	1.33	0

Table S7. Groups of representative *csp* genes in enteropathogenic *Yersinia* and *E. coli*

Group	Representative *csp* gene(s) in *Y. enterocolitica* [1]	Representative *csp* gene(s) in *Y. pseudotuberculosis* [1]	Representative *csp* gene(s) in *E. coli* [1]	Currently used name(s) [2]	RNP1 [3]	RNP2 [3]
I	*YE3821, YE3823, YE0940, YEP1_13141, YWA314_15556, DJ40_2826, DJ55_3891*	*YPTB3585,YPTB3587, YPTB2950, BZ22_1031, BZ22_1033, YpsIP31758_0374*	*b3556, b1557, b0990, b1552*	*cspA, cspB, cspG, cspI*	KGFGFI$^{T}/_{S}$P	VFVHF
II	*YE2590, YE1765, YE3012*	*YPTB2414, YPTB1624, YPTB1088*	*b1823, b0623*	*cspC, cspE*	KGFGFITP	VFVHF
III	*YE1546*	-	-	*cspE2*	KGFGFIEQ	VFVHF
IV	*YE1547, YE105_C2577*	*YPTB1423*	-	*cspB*	EGYGFISP	VYV$^{S}/_{N}$K
V	*YE1516, YEP1_01857*	*YPTB1392*	*b0880*	*cspD*	KGFGFICP	IFAHY
VI	-	-	*b1558, b0989*	*cspF, cspH*	SGKG$^{F}/_{L}$I$^{I}/_{T}$P	VQ$^{V}/_{L}$H$^{I}/_{V}$

[1] Hyphen indicates no representative *csp* gene in this species.

[2] Naming convention as in *E. coli,* with the exception of Csps in group III and IV.

[3] RNA-binding motif.

Table S8. Distribution of strains included in Csp phylogenic analysis in Enterobacterales

Family	Genus	Strain number	Csp number	Csps per strain
Enterobacteriaceae	*Candidatus Moranella*	1	2	2.0
	Candidatus Riesia	1	1	1.0
	Cedecea	1	4	4.0
	Citrobacter	4	16	4.0
	Cronobacter	6	31	5.2
	Enterobacter	6	26	4.3
	Escherichia	5	34	6.8
	Klebsiella	2	12	6.0
	Kluyvera	2	10	5.0
	Leclercia	1	4	4.0
	Raoultella	2	13	6.5
	Salmonella	2	11	5.5
	Shigella	4	22	5.5
	Shimwellia	1	5	5.0
	Yokenella	1	5	5.0
Erwiniaceae	*Buchnera*	1	2	2.0
	Erwinia	7	27	3.9
	Pantoea	5	17	3.4
	Tatumella	2	6	3.0
	Wigglesworthia	1	1	1.0
Hafniaceae	*Edwardsiella*	2	14	7.0
	Hafnia	1	7	7.0
	Obesumbacterium	1	7	7.0
Morganellaceae	*Moellerella*	2	11	5.5
	Photorhabdus	3	17	5.7
	Proteus	2	8	4.0
	Providencia	6	37	6.2
	Xenorhabdus	5	24	4.8
Pectobacteriaceae	*Brenneria*	1	3	3.0
	Dickeya	2	8	4.0
	Pectobacterium	3	12	4.0
	Sodalis	1	3	3.0
Yersiniaceae	*Rahnella*	1	6	6.0
	Serratia	7	44	6.3
	Yersinia	12	84	7.0
Total		104	534	5.1
